# Gestational Buprenorphine Exposure Disrupts Dopamine Neuron Activity and Related Behaviors in Adulthood

**DOI:** 10.1523/ENEURO.0499-21.2022

**Published:** 2022-07-27

**Authors:** Hannah B. Elam, Jennifer J. Donegan, Jenny Hsieh, Daniel J. Lodge

**Affiliations:** 1 Department of Pharmacology and Center for Biomedical Neuroscience, University of Texas Health Science Center, San Antonio, TX 78229; 2 Department of Psychiatry and Behavioral Sciences, Dell Medical School at University of Texas Austin, Austin, TX 78712; 3 Department of Neuroscience, Developmental and Regenerative Biology, University of Texas at San Antonio, San Antonio, TX 78249; 4 Brain Health Consortium, University of Texas at San Antonio, San Antonio, TX 78249; 5 Audie L. Murphy Division, South Texas Veterans Health Care System, San Antonio, TX 78229

**Keywords:** buprenorphine, development, dopamine, opioid, oxycodone

## Abstract

Opioid misuse among pregnant women is rapidly increasing in the United States. The number of maternal opioid-related diagnoses increased by 131% in the last 10 years, resulting in an increased number of infants exposed to opioids *in utero* and a subsequent increase in infants developing neonatal abstinence syndrome (NAS). The most prescribed treatment to combat maternal opioid use disorder is buprenorphine, a partial μ-opioid receptor agonist and κ-opioid receptor antagonist. Buprenorphine treatment effectively reduces NAS but has been associated with disrupted cortical development and neurodevelopmental consequences in childhood. Less is known about the long-term neurodevelopmental consequences following buprenorphine exposure *in utero*. Previous research has shown that gestational buprenorphine exposure can induce anxiety-like and depressive-like phenotypes in adult rats, suggesting that exposure to buprenorphine *in utero* may render individuals more susceptible to psychiatric illness in adulthood. A common pathology observed across multiple psychiatric illnesses is dopamine system dysfunction. Here, we administered the highly-abused opioid, oxycodone (10 mg/kg, i.p.) or a therapeutic used to treat opioid use disorder, buprenorphine (1 mg/kg, i.p) to pregnant Sprague Dawley rats from gestational day 11 through 21, then examined neurophysiological alterations in the mesolimbic dopamine system and dopamine-dependent behaviors in adult offspring. We found that gestational exposure to buprenorphine or oxycodone increases dopamine neuron activity in adulthood. Moreover, prenatal buprenorphine exposure disrupts the afferent regulation of dopamine neuron activity in the ventral tegmental area (VTA). Taken together, we posit that gestational buprenorphine or oxycodone exposure can have profound effects on the mesolimbic dopamine system in adulthood.

## Significance Statement

The opioid epidemic in the United States is a growing problem that affects people from all demographics, including pregnant women. In 2017, nearly 21,000 pregnant women reported misusing opioids during pregnancy, which can lead to many physiological and neurodevelopmental complications in infants. To combat illicit opioid use during pregnancy, buprenorphine is the priority treatment option, as it reduces illicit opioid use and alleviates symptoms of neonatal abstinence syndrome (NAS) in infants. However, less is known about the long-term neurophysiological consequences of *in utero* opioid or buprenorphine exposure. Here, we demonstrate that both oxycodone and buprenorphine exposure, *in utero*, can result in aberrant dopamine system function in adult rats. These results provide evidence of potential long-lasting effects of opioid exposure during development.

## Introduction

The opioid epidemic is an ever-growing crisis in the United States that affects people from all demographics, including pregnant women and infants. From 2010 to 2017, the number of maternal opioid-related diagnoses increased by 131%, resulting in a significant increase in the number of infants exposed to opioids *in utero* (Hirai et al., 2021). Opioid misuse is especially troubling for pregnant women, as these compounds can easily pass through the placenta and disrupt fetal development (Coyle et al., 2018). Short-term consequences of opioid misuse during pregnancy are well documented in infancy (i.e., premature birth, low birth weight, respiratory problems, and sleep deprivation; Kocherlakota, 2014; Coyle et al., 2018) and childhood (i.e., cognitive and behavioral deficits; Fill et al., 2018; Tobon et al., 2019). To combat illicit opioid misuse, buprenorphine, a partial μ-opioid receptor agonist, full κ-opioid and δ-opioid receptor antagonist, and full agonist at the nociceptin opioid peptide receptor (NOP), is the recommended pharmacological treatment option for pregnant women (Women, Acog Committee on Health Care for Underserved, and Medicine American Society of Addiction, 2012) as it provides therapeutic relief with less risk of overdose (Mozurkewich and Rayburn, 2014; Bell et al., 2009). Although buprenorphine can effectively reduce symptoms of NAS and decrease infant hospitalization (Jones et al., 2012), it also crosses the placenta (Nanovskaya et al., 2002) and has been associated with disruptions in neuronal development and cognitive and behavioral consequences in childhood, such as visual motor deficits, hyperactivity, and problems with attention, that are similar to those following illicit opioid use during pregnancy (Sanchez et al., 2008; Sundelin Wahlsten and Sarman, 2013).

The opioid epidemic began ∼20 years ago (Centers for Disease, Control, and Prevention, 2011); therefore, few studies can provide longitudinal data on the psychiatric consequences of *in utero* buprenorphine exposure in adults. Further, as these data become available, it will be difficult to disentangle the effects of prenatal opioid exposure from other childhood environmental stressors commonly associated with parental drug abuse (Ornoy et al., 2001; Davis et al., 2010). Thus, preclinical models have been used to provide insight into the long-term neurobiological consequences of prenatal opioid exposure. Specifically, prenatal buprenorphine exposure in rodents has been associated with increased depression-like and anxiety-like behavior in adulthood (Hung et al., 2013; Kongstorp et al., 2020; Schlagal et al., 2021). A common site of pathology across various psychiatric disorders is the mesolimbic dopamine system and the brain regions that can modulate dopamine neuron activity (Lodge and Grace, 2011a; Chaudhury et al., 2013; Perez and Lodge, 2013; Tye et al., 2013; Rincón-Cortés and Grace, 2017). Our laboratory has shown previously that multiple, unique disruptions to *in utero* development can lead to long-term changes in dopamine cell activity and produce behavioral alterations consistent with mental illness (Lodge and Grace, 2011a; Perez and Lodge, 2018; Donegan et al., 2020). Although illicit and prescribed opioids can cross the placenta and activate opioid receptors on midbrain dopamine neurons (Fields and Margolis, 2015), it has yet to be determined whether prenatal buprenorphine exposure renders individuals more susceptible to aberrant dopamine system function in adulthood.

Here, we modeled opioid misuse and treatment during pregnancy in Sprague Dawley rats by administration of oxycodone or buprenorphine, respectively, and used adult offspring to examine neurophysiological alterations in the mesolimbic dopamine system and dopamine-dependent behaviors. We observed that rats exposed to a commonly abused opioid, oxycodone (10 mg/kg) or the therapeutic buprenorphine (1 mg/kg), *in utero*, exhibited increases in ventral tegmental area (VTA) dopamine neuron population activity and displayed deficits in prepulse inhibition of startle (PPI). We then examined the afferent regulation of VTA dopamine neuron activity from the ventral hippocampus (vHipp) and paraventricular nucleus of the thalamus (PVT), two brain regions whose dysregulation has been implicated in psychiatric disorders (Lodge and Grace, 2007, 2011a; Perez and Lodge, 2018). We observed that inhibition of vHipp or PVT activity in oxycodone-treated but not buprenorphine-treated animals, restored dopamine system function. These findings suggest that prenatal exposure to buprenorphine can produce enduring neural circuit alterations and behavioral deficits associated with dysfunction of the dopamine system, which may have important implications for the development of mental illness.

## Materials and Methods

All experiments were performed in accordance with the guidelines outlined in the United States Public Health Service Policy *Guide for the Care and Use of Laboratory Animals* and were approved by the Institutional Animal Care and the Use Committees of University of Texas Health San Antonio and United States Department of Veterans Affairs.

### Animals

Rats were maintained in a temperature-controlled environment, on a 12/12 h light/dark cycle, with *ad libitum* access to food and water. Male and female rats were used in this study and both pooled and disaggregated data are presented in the results.

### Drug administration

**Figure 1.**
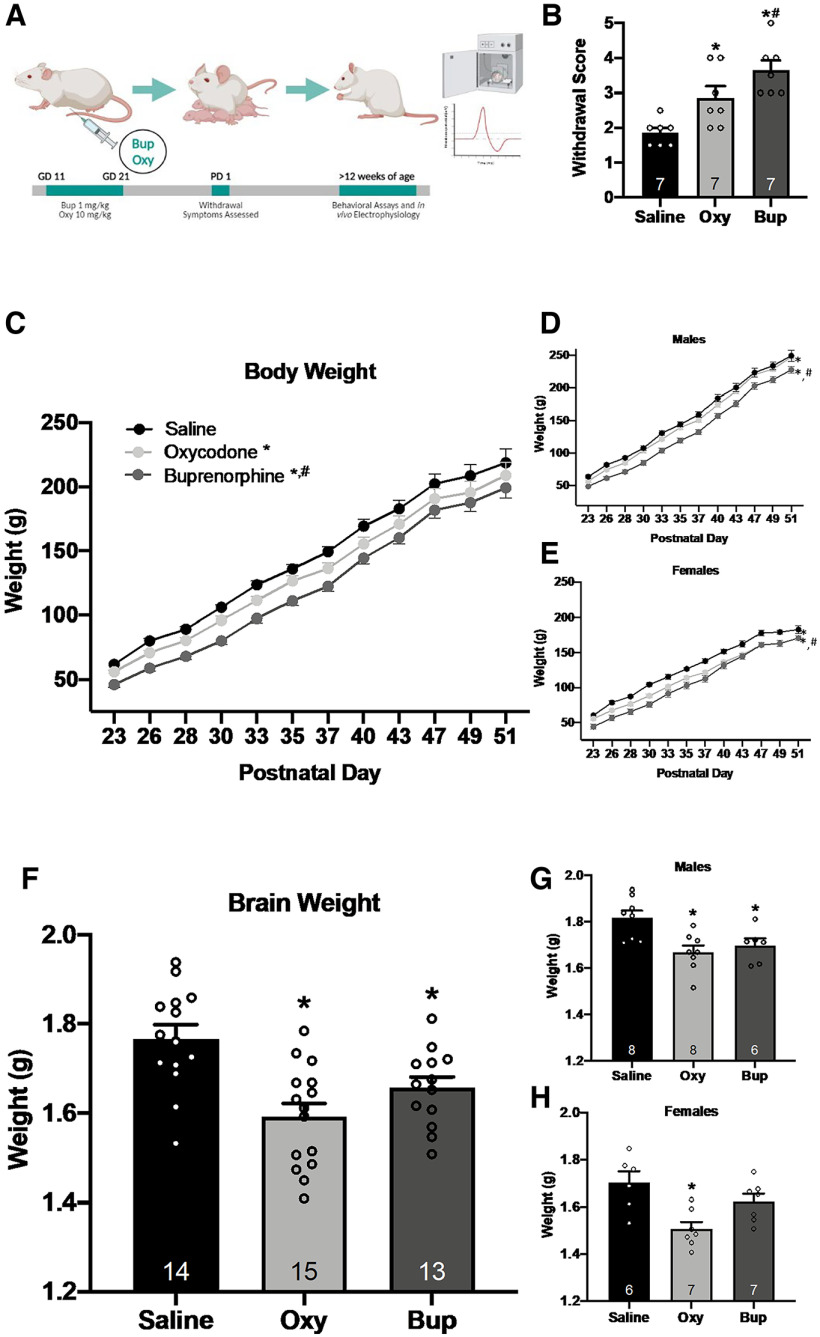
Gestational opioid exposure results in physiological alterations that persist throughout adulthood. ***A***, Schematic representation of gestational opioid exposure. Pregnant rats received intraperitoneal injections of either buprenorphine (1 mg/kg), oxycodone (10 mg/kg), or saline from gestational day (GD) 11 through 21. Withdrawal symptoms were assessed on postnatal day (PD) 1. Rats were weaned on PD 21 then PPI and VTA dopamine neuron activity were assessed in adulthood (>12 weeks of age). ***B***, Pups exposed to oxycodone and buprenorphine *in utero* displayed more withdrawal behavior than saline-treated pups; *p < 0.05 compared with saline. *n *=* *7 rats per group. Additionally, gestational buprenorphine exposure resulted in more withdrawal behavior than gestational oxycodone exposure; #*p* < 0.05 compared with oxycodone. *n *=* *7 rats per group. ***C***, Body weight from weaning until adulthood was significantly reduced in oxycodone-treated and buprenorphine-treated animals; *p < 0.05 compared with saline, *n *=* *13–16 rats per group. Further, buprenorphine rats displayed lower body weight than oxycodone rats; #*p* < 0.05 compared with oxycodone, *n *=* *16 rats per group. This trend was the same in males (***D***) and females (***E***). ***F***, Brain weight was measured in adulthood. Adult rats exposed to gestational oxycodone or buprenorphine exhibited lower brain weight compared with saline controls; *p < 0.05 compared with saline, *n *=* *13–15 rats per group. The same trend was observed in adult males (***G***) but not adult females (***H***). Females exposed to oxycodone but not buprenorphine had lower brain weight in adulthood; *p < 0.05 compared with saline, *n *=* *6–7 rats per group.

Pregnant Sprague Dawley rats (250–275 g) were obtained from Envigo RMS Inc. on gestational day 9. To model chronic opioid use during pregnancy, pregnant dams received daily intraperitoneal injections of clinically relevant doses (Hutchings et al., 1996; Robinson and Wallace, 2001; Griffin et al., 2019) of either oxycodone (10 mg/kg), buprenorphine (1 mg/kg), or saline (1 ml/kg) from gestational day 11 through 21 (Fig. 1*A*). Pups were weaned on postnatal day 21 and housed with littermates in groups of two to three rats per cage. Behavioral and electrophysiological experiments were conducted in adult animals (>12 weeks of age). Offspring from multiple litters were used for all experiments.

### Withdrawal

Symptoms of withdrawal were assessed in male and female pups on postnatal day 1. Pups were placed in an observation chamber maintained at 34.5°C. Behavior was observed and videotaped for 10 min and scored by two experimenters blinded to treatment conditions. The presence or absence of the following withdrawal behaviors was recorded: vocalization, hyperactivity, stretching, face washing, and tremors (Griffin et al., 2019; Wallin et al., 2019). A score of 1 was given for each behavior exhibited by the pup. The absence of a behavior was scored as 0. The final sum of each behavioral score was used to determine the final withdrawal score between 0 and 5, with 5 being the most withdrawal symptoms.

### Body and brain weight

Physiologic assessments of body weight and brain weight were performed in oxycodone-treated, buprenorphine-treated, and saline-treated offspring. Body weight of the pups was measured from postnatal day 23 to 51. Brain weight was assessed in adult rats (>12 weeks of age), immediately following transcardial perfusion.

### PPI response

Adult rats that were exposed to either prenatal buprenorphine, oxycodone, or saline were placed in a sound attenuated chamber (SD Instruments) and allowed to acclimate for 5 min to 65 dB background noise. Following acclimation, rats were exposed to ten startle-only trials [40 ms, 120 dB, 15-s average intertrial intervals (ITIs)]. Rats were then exposed to 24 trials where a prepulse (20 ms at 69, 73, or 81 dB) was presented 100 ms before the startle pulse. Each prepulse + startle pulse combination was presented in a pseudo-random order six times (15-s average ITI). The startle response was measured from 10 to 80 ms after the onset of the startle only pulse and recorded using SR-LAB Analysis Software (SD Instruments). PPI was calculated as a percentage score for each prepulse intensity using the following formula: %PPI = (100 × [(pulse alone score) – (prepulse + pulse score)/(pulse alone score)].

### *In vivo* extracellular dopamine neuron recordings

Adult rats were anesthetized with 8% chloral hydrate (400 mg/kg, i.p.) and placed in a stereotaxic apparatus. Chloral hydrate was used for all dopamine recordings to avoid significantly depressing dopamine neuron activity (Hyland et al., 2002). Supplemental anesthesia was administered to maintain suppression of limb compression withdrawal reflex and core body temperature of 37°C was sustained using a thermostatically controlled heating pad (PhysioSuite, Kent Scientific Coorporation). Extracellular glass microelectrodes (impedance ∼6–10 MΩ) were lowered into the VTA (A/P 5.3–5.7, M/L 0.6–1.0 from bregma, D/V −6.5 to −9.0 mm from the brain surface) using a hydraulic micropositioner (Model 640, Kopf Instruments). Three parameters of dopamine activity were measured and analyzed: the number of dopamine neurons firing spontaneously (population activity; Lodge and Grace, 2011a), basal firing rate, and proportion of action potentials occurring in bursts. Spontaneously active dopamine neurons were recorded using previously established electrophysiological criteria (Grace and Bunney, 1983; Ungless and Grace, 2012b). In short, dopamine neurons are identified by biphasic action potentials, >2.0 ms in duration (Ungless and Grace, 2012a). Dopamine neurons in the VTA were identified and recorded for 3–5 min (low-frequency cutoff: 30 Hz; high-frequency cutoff: 30 kHz). A predetermined pattern of six to nine vertical passes, separated by 200 μm, was used to sample various regions of the VTA. Electrophysiological analysis of dopamine neuron activity was performed using commercially available computer software (LabChart version 8; ADInstruments). Single-cell extracellular recordings lasted no longer than 2 h.

### Intracranial drug administration

A subset of adult rats received intracranial administration of tetrodotoxin citrate (TTX; 0.25 ng in 0.75 μl) directly into the vHipp (A/P −5.3, M/L ±5.0, D/V −6.5) or PVT (A/P −2.0, M/L ±0.6, D/V −4.3). Intracranial administration was performed using a standard guide cannula (26 gauge; Plastics One) with an internal cannula extending 1 mm past the end of the guide. Recordings began ∼10 min following TTX injection and lasted no longer than 2 h after TTX administration.

### Histology

Rats were transcardially perfused immediately following all electrophysiological recordings with saline (150 ml) followed by formaldehyde (150 ml; 4% w/v in PBS), then rapidly decapitated. Brains were postfixed for at least 24 h (4% formaldehyde in saline) and cryoprotected (10% w/v sucrose in PBS) until saturated. Histologic verification of electrode tracks within the VTA and correct cannula placement in either the vHipp or PVT were performed in coronal sections; 25-μm sections were collected on a cryostat (Leica) and mounted onto gelatin-chrom-coated slides and stained with neutral red (0.1%) and thionin acetate (0.01%; Paxinos and Watson, 1998).

### Statistical analysis

Body weight data were analyzed using a two-way ANOVA (prenatal treatment × postnatal day). Brain weight, withdrawal scores, and electrophysiological data were analyzed using a one-way ANOVA. PPI data were analyzed by two-way ANOVA (prenatal treatment × dB). Holm–Sidak or Dunn’s method (if data failed test for normality and/or equal variance) were used for *post hoc* comparisons. All data are represented as the mean ± SEM, unless otherwise stated, with n values representing the number of rats per group unless otherwise specified. Significance was determined at *p* < 0.05. All data were analyzed using SigmaPlot (Systat Software Inc.) and graphed using Prism software (GraphPad Software Inc.).

### Materials

Buprenorphine (item #14025; Cayman Chemical) and Oxycodone (item #O1169; Spectrum Chemicals) were made fresh daily and dissolved in saline. TTX was purchased from Tocris Bioscience (catalog #1069). Chloral hydrate (item #C8383) was purchased from Sigma-Aldrich. All other chemicals and reagents were of either analytical or laboratory grade and purchased from standard suppliers.

## Results

### Withdrawal score

Prenatal exposure to an opioid has been shown to produce opioid dependence in both humans and rodents (Kocherlakota, 2014; Coyle et al., 2018; Griffin et al., 2019; Hirai et al., 2021). Consistent with previous literature (Griffin et al., 2019; Wallin et al., 2019), gestational exposure to oxycodone (Fig. 1*B*; one-way ANOVA; *F*_(2,20)_ = 11.775; *p* < 0.001; Holm–Sidak: *t *=* *2.711, *p *=* *0.028) or buprenorphine (*t *=* *4.4841, *p* < 0.001) was found to induce significantly more withdrawal behavior compared with offspring with gestational exposure to saline. Further, offspring exposed to prenatal buprenorphine exhibited a significantly greater withdrawal response than offspring exposed to oxycodone (*t *=* *2.130, *p *=* *0.047).

### Body and brain weight

Physiologic characteristics of gestational opioid exposure are well characterized in rodents (Wallin et al., 2019; Griffin et al., 2019) and include decreased body weight and brain volume. Consistent with previous literature (Griffin et al., 2019; Wallin et al., 2019), we found a main effect of prenatal treatment in male and female offspring combined (Fig. 1*C*; two-way ANOVA; factors: prenatal treatment × postnatal day; *F*_(2,539)_ = 52.539; *p* < 0.001, *n *=* *13–16 rats/group), demonstrating gestational opioid exposure significantly reduces offspring body weight. Oxycodone-treated (Holm–Sidak: *t *=* *4.862, *p* < 0.001, *n *=* *16 rats) and buprenorphine-treated (Holm–Sidak: *t *=* *10.209, *p* < 0.001, *n *=* *16 rats) offspring weighed significantly less than saline offspring (*n *=* *13 rats). Further, body weight was significantly lower in buprenorphine-treated offspring, compared with oxycodone-treated offspring (Holm–Sidak: *t *=* *5.647, *p* < 0.001). However, the rate of change in body weight for oxycodone and buprenorphine offspring did not vary from controls (*p *=* *0.9351). When male and female offspring weights were separated, oxycodone males (Fig. 1*D*; two-way ANOVA; *F*_(2,275)_ = 100.189; *p* < 0.001; Holm–Sidak: *t *=* *3.624, *p* < 0.001, *n *=* *8 rats), oxycodone females (Fig. 1*E*; two-way ANOVA; *F*_(2,263)_ = 95.610; *p* < 0.001; Holm–Sidak: *t *=* *9.096, *p* < 0.001, *n *=* *8 rats), buprenorphine males (Holm–Sidak: *t *=* *13.508, *p* < 0.001, *n *=* *8 rats) and buprenorphine females (Holm–Sidak: *t *=* *13.745, *p* < 0.001, *n *=* *8 rats) exhibited reduced body weight compared with saline controls (males: *n *=* *7 rats; females: *n *=* *6 rats). Buprenorphine treatment also significantly reduced body weight compared with oxycodone treatment in both male (Holm–Sidak: *t *=* *10.231, *p* < 0.001) and female (Holm–Sidak: *t *= 5.022, *p* < 0.001) offspring.

In addition to decreased body weight, prenatal opioid exposure has been shown to result in decreased brain mass in adolescence (Hung et al., 2013). Here, we examined changes in brain weight in adulthood, following prenatal exposure to either oxycodone or buprenorphine. When combining male and female data, there was a significant main effect of prenatal treatment (Fig. 1*F*; one-way ANOVA; *F*_(2,41)_ = 9.955; *p* < 0.001, *n *=* *13–15 rats/group) on adult brain weight. Oxycodone-treated (Holm–Sidak: *t *= 4.427, *p* < 0.001, *n *=* *15 rats) and buprenorphine-treated (Holm–Sidak: *t *=* *2.690, *p *=* *0.021, *n *=* *13 rats) rats exhibited lighter brain weights than saline-treated rats (*n *=* *14 rats). When disaggregated by sex, males alone displayed a similar trend in brain weight (Fig. 1*G*; one-way ANOVA; *F*_(2,21)_ = 6.827; *p *=* *0.006), with oxycodone-treated males (Holm–Sidak: *t *=* *3.521, *p *=* *0.007, *n *=* *8 rats) and buprenorphine-treated males (Holm–Sidak: *t *= 2.624, *p *=* *0.033, *n *=* *6 rats) displaying significantly lighter brain weights than saline-treated males (*n *=* *8 rats). However, in females, only oxycodone-treated rats displayed a significant decrease in brain weight (Fig. 1*H*; one-way ANOVA; *F*_(2,19)_ = 7.366; *p *=* *0.005) compared with saline-treated females (Holm–Sidak: *t *=* *3.790, *p *= 0.004, oxycodone: *n *=* *7 rats; saline: *n *=* *6 rats), while buprenorphine-treated rats were not statistically different from controls (Holm–Sidak: *t *=* *1.531, *p *=* *0.144, *n *=* *7 rats).

### PPI

**Figure 2.**
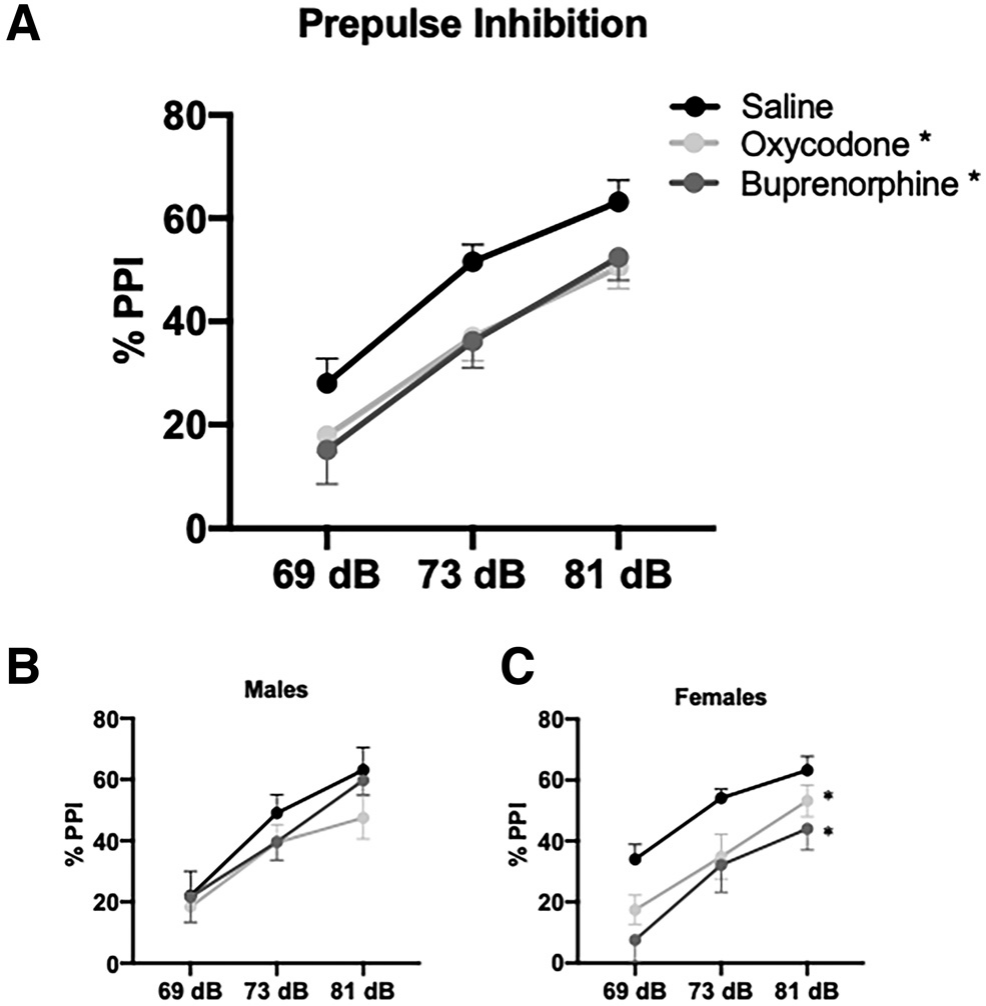
Prenatal opioid exposure results in PPI deficits in adulthood. Gestational exposure to oxycodone or buprenorphine produces adulthood deficits in PPI in male and female rats combined; *p < 0.05 compared with saline, *n *=* *15–17 rats per group (***A***). When disaggregated by sex, there were no significant differences between the males (***B***). However, oxycodone-treated and buprenorphine-treated females (***C***) had significant deficits in PPI compared with saline; *p < 0.05 compared with saline, *n *=* *7–9 rats per group.

Sensorimotor gating is the ability to filter irrelevant sensory information and it is commonly disrupted across a range of psychiatric disorders. PPI is an assay used to measure sensorimotor gating in both humans and rodent models. Consistent with previous literature, a main effect of prepulse intensity was observed. As the prepulse magnitude increased, the startle response was attenuated (Fig. 2*A*; two-way ANOVA; *F*_(2,143)_ = 45.054; *p* < 0.001; *n *=* *15–17 rats/group). A main effect of prenatal treatment was also observed (two-way ANOVA; factors: prenatal treatment × prepulse intensity; *F*_(2,143)_ = 7.837; *p* < 0.001) demonstrating prenatal opioid-induced deficits in PPI at all prepulse intensities measured. Rats exposed to prenatal buprenorphine (Holm–Sidak: *t *=* *3.455, *p *=* *0.002) and prenatal oxycodone (Holm–Sidak: *t *=* *3.400, *p *=* *0.002) displayed significantly greater deficits in PPI compared with rats exposed to prenatal saline. No differences were detected between buprenorphine-treated or oxycodone-treated rats (Holm–Sidak: *t* = 0.0162, *p *=* *0.871). When disaggregated by sex (Fig. 2*B*,*C*), there were no significant differences between any of the male groups (*n *=* *8 rats/group; *p *=* *0.206). However, females treated with buprenorphine (Holm–Sidak; *t *=* *4.210; *p* < 0.001; *n *=* *7 rats) or oxycodone (Holm–Sidak; *t *=* *3.037; *p *=* *0.007; *n *=* *9 rats) show significant deficits in PPI compared with saline-treated females (*n *=* *8). Taken together, these results suggest prenatal exposure to oxycodone or buprenorphine can disrupt sensorimotor gating in adulthood in a sex-specific manner.

### Dopamine neuron activity

**Figure 3.**
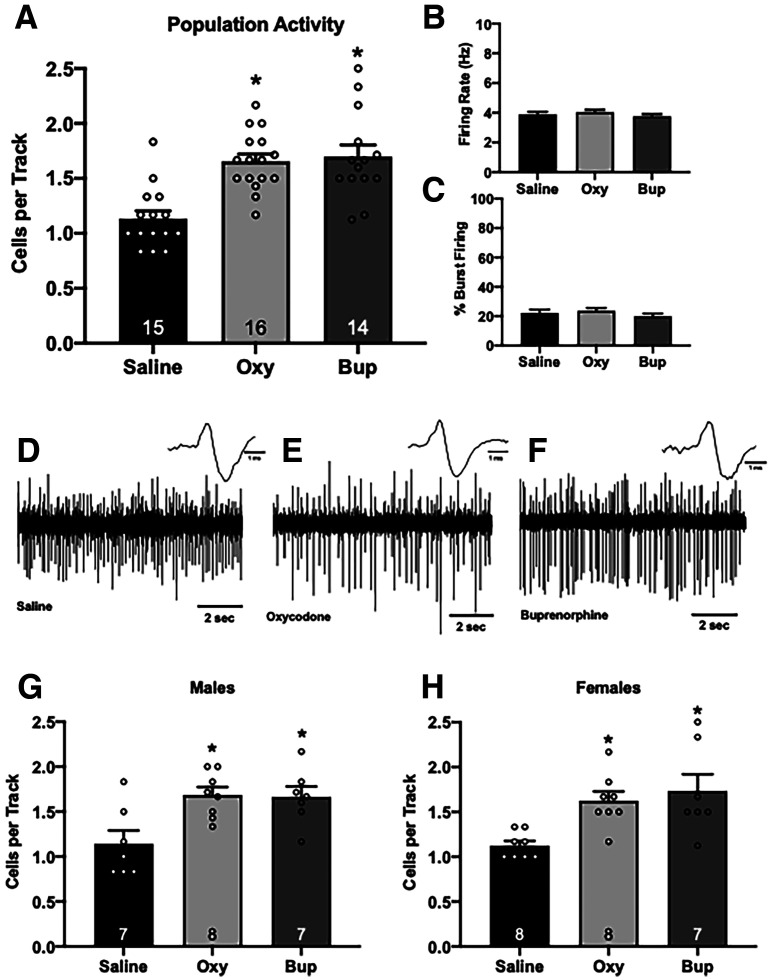
Prenatal opioid exposure significantly increases dopamine neuron population activity in adulthood. Three parameters of dopamine neuron activity were measured in male and female rats combined: (***A***) population activity (average number of spontaneously active dopamine neurons per electrode track), (***B***) average firing rate, and (***C***) average percentage of spikes firing in a burst. Gestational oxycodone or buprenorphine exposure increases dopamine population activity in adulthood (***A***): *p < 0.05 compared with saline. There were no significant changes in firing rate (***B***) or bursting pattern (***C***). Representative traces from saline animals (***D***) oxycodone animals (***E***) and buprenorphine animals (***F***) *n *= 14–16 rats per group. Increased population activity was observed in males (***G***) and females (***H***) exposed to oxycodone or buprenorphine *in utero*; *p < 0.05 compared with saline.

Dopamine dysregulation is seen across a wide range of psychiatric illnesses and can be measured in rodents using *in vivo* extracellular electrophysiology. Consistent with other rodent models of psychiatric disorders, prenatal buprenorphine (Fig. 3*A*; one-way ANOVA; *F*_(2,44)_ = 14.791; *p* < 0.001; Holm–Sidak: *t* = 4.814, *p* < 0.001, *n *=* *14; 1.70 ± 0.11 cells/track) and oxycodone (Holm–Sidak: *t *=* *4.596, *p* < 0.001, *n *=* *16; 1.66 ± 0.07 cells/track) treatment resulted in a significant increase in VTA dopamine neuron population activity compared with saline controls (*n *=* *15; 1.13 ± 0.07 cells/track). No significant differences were detected between groups in the average firing rate (Fig. 3*B*; saline = 102 cells; 3.89 ± 0.18 Hz; oxycodone = 161 cells; 4.04 ± 0.16 Hz; buprenorphine = 144 cells; 3.76 ± 0.15 Hz) or percentage of cells burst firing (Fig. 3*C*; saline = 102 cells; 22.18 ± 2.38%; oxycodone = 161 cells; 23.68 ± 1.95%; buprenorphine = 144 cells; 19.99 ± 1.81%). Representative traces are shown in Figure 3*D–F*. Similar results were obtained in both male (Fig. 3*G*) and female (Fig. 3*H*) rats. Males exposed to prenatal oxycodone (one-way ANOVA; *F*_(2,21)_ = 6.105; *p *=* *0.009; Holm–Sidak: *t *=* *3.123, *p *=* *0.017, *n *=* *7 rats; 1.71 ± 0.10 cells/track) or buprenorphine (Holm–Sidak: *t *=* *2.843, *p *=* *0.021, *n *=* *7 rats; 1.66 ± 0.12 cells/track) had an increase in population activity compared with saline-treated controls (*n *=* *8 rats; 1.19 ± 0.14 cells/track). Similarly, female offspring treated with oxycodone (one-way ANOVA; *F*_(2,22)_ = 7.255; *p *=* *0.004; Holm–Sidak: *t *=* *2.992, *p *=* *0.014, *n *=* *8 rats; 1.63 ± 0.10 cells/track) or buprenorphine (Holm–Sidak: *t *=* *3.509, *p *=* *0.007, *n *=* *7 rats; 1.73 ± 0.19 cells/track) also displayed significantly greater dopamine neuron population activity compared with saline females (*n *=* *8; 1.13 ± 0.05 cells/track). When disaggregated by sex, there were no significant effects of prenatal opioid in average firing rate or percentage of cells burst firing (data not shown).

### Afferent regulation of dopamine neuron activity

**Figure 4.**
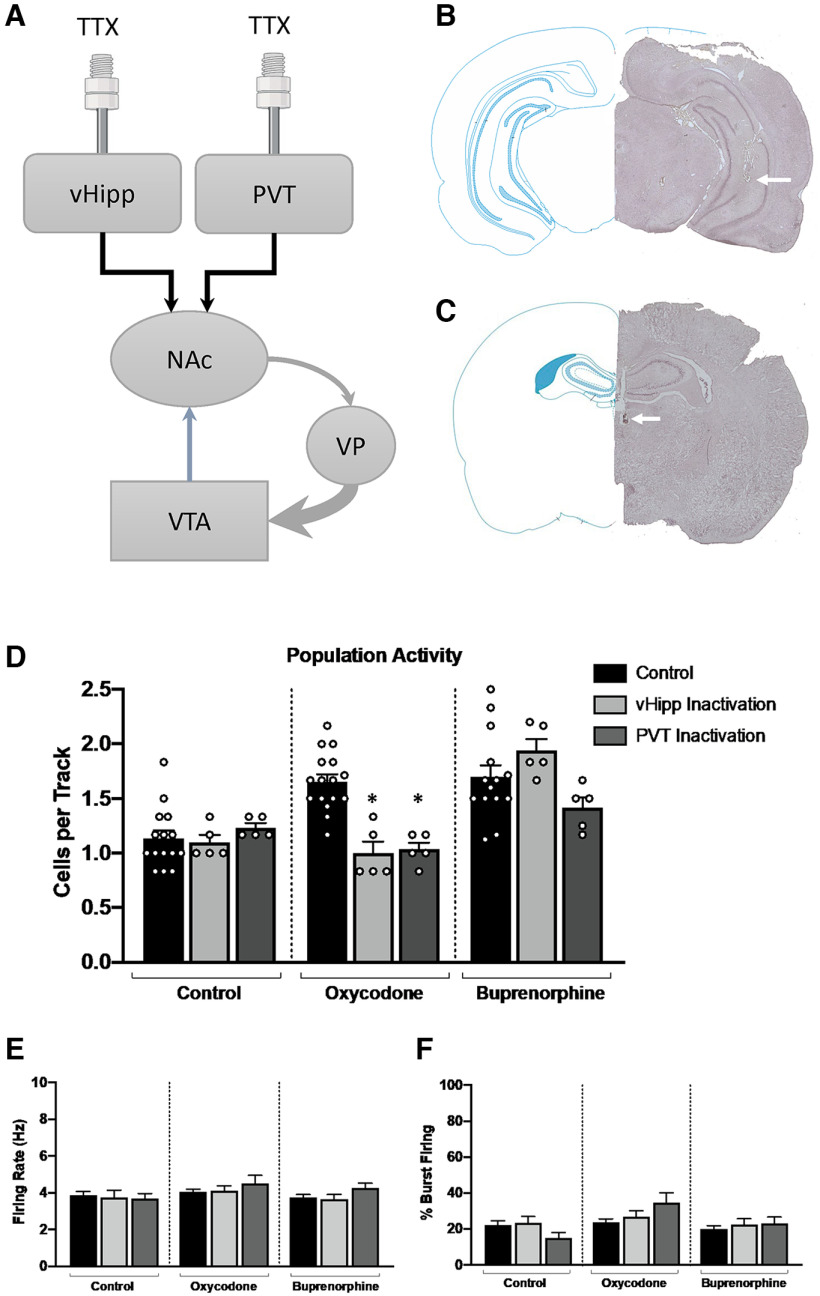
Inhibition of afferent regulation of dopamine neuron activity does not reverse population activity in buprenorphine-treated adults. ***A***, Schematic representation of the multisynaptic circuit by which the vHipp and PVT regulate dopamine activity in the VTA. In healthy animals, GABAergic projections from the ventral pallidum (VP) provide tonic inhibition of VTA dopamine neuron activity. In animal models used to study psychosis, increased activity of glutamatergic inputs from the vHipp or PVT to the NAc result in downstream hyperfunction of the dopamine system which can be reversed by selective injection of TTX. Representative cannula tracks, indicated by an arrow, in the vHipp (***B***) and PVT (***C***) and corresponding schematics of the brain section. ***D***, Dopamine system function was restored following TTX inactivation of either the vHipp or PVT in oxycodone-treated animals but not buprenorphine-treated animals; *p < 0.05 denotes significant difference from control oxycodone animals. There were no changes in the firing rate (***E***) or percentage of cells bursting (***F***) in any treatment.

While dysregulation of the mesolimbic dopamine system is commonly observed in a variety of psychiatric illnesses, no obvious histopathology has been identified in the VTA. Rather, the pathology appears to lie in the brain regions that regulate dopamine neuron activity. To examine potential afferents contributing to the dysregulation of VTA dopamine neuron activity, we injected TTX into either the vHipp or PVT, two regions known to regulate dopamine neuron population activity (Fig. 4*A–C*). Consistent with previous research in models of dopamine hyperfunction (Perez and Lodge, 2018), we found that TTX inactivation of either the vHipp (Fig. 4*D*; one-way ANOVA; *F*_(2,25)_ = 21.26; *p* < 0.001; Holm–Sidak: *t *=* *5.263, *p* < 0.001, *n *=* *5 rats; 1.00 ± 0.21 cells/track) or PVT (Holm–Sidak: *t* = 4.994, *p* < 0.001, *n *=* *5 rats; 1.03 ± 0.06 cells/track) reversed aberrant dopamine neuron population activity in oxycodone offspring, compared with oxycodone baseline (*n *=* *16; 1.66 ± 0.07). However, TTX inactivation of the vHipp (Fig. 4*D*; one-way ANOVA; *F*_(2,21)_ = 2.947; *p *=* *0.075; Holm–Sidak: *t *=* *1.360, *p *=* *0.2408, *n *=* *5 rats; 1.94 ± 0.10 cells/track) or PVT (Holm–Sidak: *t *=* *1.582, *p *=* *0.2408, *n *=* *5 rats; 1.42 ± 0.09 cells/track) was not able to reverse aberrant dopamine neuron activity observed in buprenorphine rats (*n *=* *14; 1.70 ± 0.11 cells/track). Importantly, TTX inactivation of the vHipp (Fig. 4*D*; one-way ANOVA; *F*_(2,22)_ = 0.47; *p *=* *0.353; Holm–Sidak: *t *=* *0.277, *p *=* *0.7841, *n *=* *5 rats; 1.1 ± 0.07 cells/track) or PVT (Holm–Sidak: *t *=* *0.834, *p *=* *0.7546, *n *=* *5 rats; 1.23 ± 0.04 cells/track) did not significantly alter population activity in control animals. No significant differences in firing rate or the percentage of cells burst firing were detected between any of the groups (Fig. 4*E*,*F*).

## Discussion

In the current experiments, we modeled prenatal opioid exposure in Sprague Dawley rats to demonstrate that buprenorphine exposure *in utero* can result in aberrant dopamine system function in adulthood. Buprenorphine, a partial μ-opioid receptor agonist, full κ-opioid and δ-opioid receptor antagonist, and full agonist at the NOP (Kumar et al., 2014; Madison and Eitan, 2020), is the preferred treatment for opioid use disorder during pregnancy (Bell et al., 2009; Women, Acog Committee on Health Care for Underserved, and Medicine American Society of Addiction, 2012; Mozurkewich and Rayburn, 2014). Maternal treatment with buprenorphine during pregnancy effectively reduces neonatal abstinence syndrome (NAS) and time spent in the hospital (Jones et al., 2010), but gestational buprenorphine exposure has also been shown to result in childhood neurodevelopmental consequences similar to those of illicit opioid use during pregnancy (Sundelin Wahlsten and Sarman, 2013; Sanchez et al., 2008).

Opioid misuse during pregnancy can have detrimental results not only for the mother but for the infant. Infants exposed to opioids during pregnancy typically develop NAS hours after birth, which is characterized by hyperactivity of the nervous system (Reddy et al., 2017). Common signs of NAS include irritability, tremors, sleep disturbances, and lower body weight because of poor feeding and gastrointestinal issues (Reddy et al., 2017). Although treatment with buprenorphine during pregnancy can effectively reduce signs of NAS, it does not eliminate them. Rather, buprenorphine exposure *in utero* reduces treatment duration and amount of medication used to treat symptoms of NAS (Hall et al., 2016; Kraft et al., 2017). Consistent with previous literature (Griffin et al., 2019; Wallin et al., 2019), we found that prenatal exposure to either buprenorphine or oxycodone resulted in significant withdrawal symptoms immediately after birth. Further, treatment with oxycodone or buprenorphine was associated with lower body weight from weaning until adulthood, in both male and female offspring. There were no differences in the growth rate between groups, suggesting that opioid exposure *in utero* does not disrupt the rate of weight change across time but instead decreases the baseline body weight in oxycodone and buprenorphine offspring. In addition to lower body weight, offspring exposed to gestational oxycodone or buprenorphine exhibited lower brain weight in adulthood, compared with saline controls.

The prevalence of opioid use during pregnancy is relatively similar across trimesters, suggesting that the timing and duration of exposure may have differential effects on the offspring (Zhao et al., 2021). One caveat of studying prenatal opioid exposure in rodents is that the third trimester in humans corresponds to the first two weeks of postnatal life in rats (Zeiss, 2021). While many neurodevelopmental milestones occur postbirth in rats (Zeiss, 2021), the timing of our manipulation was selected to coincide with previous studies in which developmental disruptions between gestational day 11 and 21 produce robust neuropsychiatric alterations in adulthood, including autism-like and schizophrenia-like phenotypes (Lodge, 2013; Perez et al., 2014; Donegan et al., 2018, 2020). Indeed, lower total brain volume has been observed in rodent models of autism and early-life stress (Golden et al., 2021; Reshetnikov et al., 2021). Further, loss of brain volume in specific brain regions is observed in a variety of psychiatric illnesses (Angelescu et al., 2021; Steuber et al., 2021; Su et al., 2021). Previous studies have shown similar decreases in brain weight in males and female rats, following buprenorphine exposure but these results compared brain weight on postnatal day 21, not in adulthood (Hung et al., 2013). These data provide further support that prenatal exposure to buprenorphine can produce persistent physiological and neurodevelopmental alterations similar to oxycodone, a commonly abused opioid.

To assess sensorimotor gating in adult rats exposed to opioids *in utero*, we used the PPI assay, which examines the ability to filter extraneous sensory information and is commonly disrupted across multiple psychiatric disorders. Further, substance use disorder, including long-term misuse of μ opioid receptor agonists, can alter dopamine system function and subsequently disrupt sensorimotor gating (Meng et al., 2010; Lee et al., 2017). PPI is used in both humans and rodents and is associated with dopamine release in the nucleus accumbens (NAc; Braff and Geyer, 1990; Swerdlow et al., 2001). Here, we found that gestational exposure to oxycodone or buprenorphine resulted in PPI deficits in adulthood. Interestingly, previous studies have shown that chronic exposure to buprenorphine in adulthood does not impair PPI (Shafiei et al., 2019), suggesting that the timing of buprenorphine exposure can differentially alter dopamine system function. Specifically, gestational buprenorphine exposure may alter neurodevelopment and contribute to deficits in sensorimotor gating later in life. While the combined male and female data show robust deficits in PPI, this trend was primarily driven by the females. Buprenorphine and oxycodone females had profound deficits in sensorimotor gating while buprenorphine and oxycodone males were relatively less effected. Although PPI is dependent on dopamine release in the NAc, regulation of PPI is not exclusive to this region. Multiple brain regions, including the hippocampus and medial prefrontal cortex (mPFC) play a role in facilitating PPI (Swerdlow et al., 2001). It is possible buprenorphine exposure has sex-specific effects on hippocampal and PFC development and function. Indeed, recent research has demonstrated sex-specific neurodevelopmental and pharmacokinetic differences in response to opioid-receptor ligands (Kumar et al., 2021). For example, the timing of developmental myelination is preferentially affected by μ-opioid and nociception-receptor signaling in female rats (Mohamed et al., 2020). Further, systemic exposure to oxycodone is greater in adult female rats (Chan et al., 2008); however, the sex-specific alterations of hippocampal and PFC development following buprenorphine exposure remains to be elucidated. Future studies will also examine depression and reward-related behavioral outputs to further characterize the neurodevelopmental consequences of *in utero* buprenorphine exposure.

Dopamine system dysfunction has been observed across a variety of psychiatric illnesses, including depression, schizophrenia, and substance use disorder. Specifically, dopamine neuron activity in the VTA is decreased in preclinical models of depression (Chaudhury et al., 2013; Tye et al., 2013; Rincón-Cortés and Grace, 2017) and increased in preclinical models used to study schizophrenia (Lodge and Grace, 2011a; Perez and Lodge, 2013). Previous research has shown that gestational buprenorphine exposure can induce anxiety-like and depressive-like phenotypes in rats (Hung et al., 2013), suggesting that exposure to buprenorphine *in utero* can render individuals more susceptible to psychiatric illness in adulthood. In the current study, we used *in vivo* extracellular electrophysiology to examine how gestational exposure to oxycodone or buprenorphine affected VTA dopamine neuron population activity in adulthood. The number of spontaneously active dopamine neurons, in healthy animals, can be modulated to respond appropriately to various environmental stimuli. However, in rodent models used to study psychosis, the number of spontaneously active dopamine neurons is constantly elevated, resulting in constant, maximal dopamine output and the inability to distinguish between relevant and extraneous stimuli (Lodge and Grace, 2011a). Interestingly, we found that prenatal buprenorphine or oxycodone exposure significantly increased VTA dopamine neuron population activity in adult rats, similar to increases observed across multiple rodent models used to study psychosis. While we observed increases in VTA population activity, there were no changes in the firing rate or percentage of cells bursting, which is consistent with what has been previously demonstrated in rodent models of psychosis (Lodge and Grace, 2011a; Perez and Lodge, 2013, 2018; Donegan et al., 2017). Taken together, these data suggest gestational buprenorphine or oxycodone exposure can have profound effects on the mesolimbic dopamine system in adulthood. Future studies will aim to address whether there is a safe window or dose of buprenorphine during pregnancy that does not result in aberrant dopamine system function in adulthood.

Although dopamine system dysfunction is observed across multiple animal models used to study psychosis (Lodge and Grace, 2008, 2009; Perez and Lodge, 2013; Aguilar et al., 2014; Perez et al., 2016; Donegan et al., 2019), no obvious histopathology has been observed in the dopamine neurons themselves. Rather, the pathology appears to lie in upstream brain regions that regulate dopamine system function (Gill et al., 2011; Lodge and Grace, 2011b; Boley et al., 2014; Donegan et al., 2017; Perez and Lodge, 2018, 2021; Donegan et al., 2019). To assess afferent regulation of the VTA dopamine neurons following *in utero* opioid exposure, we selectively inactivated the vHipp or PVT, which are two brain regions that regulate VTA dopamine neuron population activity through a multisynaptic circuit (Fig. 4*A*) that begins with projections to the NAc (Lodge and Grace, 2011a; Perez and Lodge, 2018). Importantly, increased activity in glutamatergic projections from the vHipp and PVT to the NAc has been observed in a variety of rodent models of psychiatric disorders, including PTSD (Elam et al., 2021) and schizophrenia (Perez and Lodge, 2013; Boley et al., 2014), and this hyperactivity results in increased dopamine neuron population activity in the VTA. Previous research has demonstrated that selective inactivation of the vHipp or PVT in rodent models used to study psychosis can restore normal dopamine system function (Lodge and Grace, 2007; Perez and Lodge, 2018). To elucidate a possible mechanism by which population activity was increased in buprenorphine-treated or oxycodone-treated rats, we injected TTX into the vHipp or PVT and examined dopamine neuron population activity. TTX was used specifically to identify potential brain regions contributing to aberrant dopamine system function as it produces robust suppression of afferent neuronal activity and data obtained with this approach are consistent with more specific techniques such as chemogenetics (Lodge and Grace, 2007; Perez and Lodge, 2018; Donegan et al., 2019). Indeed, when TTX was injected into the vHipp or PVT in oxycodone-treated animals, dopamine system function was restored, consistent with previous studies in other models used to study psychiatric illness (Lodge and Grace, 2007; Perez and Lodge, 2018). Surprisingly, we found that inactivation of either the vHipp or PVT had no effect on population activity in buprenorphine-treated rats, suggesting distinct mechanisms by which developmental oxycodone and buprenorphine increase VTA dopamine neuron population activity. While both oxycodone and buprenorphine target the μ opioid receptor, we have recently demonstrated, in a human induced pluripotent stem cell (iPSC) organoid model, that developmental exposure to buprenorphine, but not oxycodone, can disrupt interneuron migration and cortical network activity through activation of the nociception receptor (NOP; Nieto-Estévez et al., 2022). Future studies will be necessary to elucidate the exact mechanism and neural circuitry contributing to the increase in dopamine neuron population activity, following prenatal buprenorphine exposure.

The consequences of opioid misuse during pregnancy are clear. Infants exposed to opioids *in utero* are at risk for immediate and long-term physiological and neurologic complications (Kocherlakota, 2014; Coyle et al., 2018; Hirai et al., 2021). Buprenorphine can reduce illicit opioid use in pregnant women and improve immediate symptoms of neonatal withdrawal but there is increasing evidence that *in utero* buprenorphine exposure may also have long-term neurodevelopmental consequences. Here, we demonstrated that gestational exposure to buprenorphine or oxycodone results in aberrant dopamine system function and deficits in sensorimotor gating in adulthood. Further, the mechanism by which *in utero* oxycodone or buprenorphine exposure increased population activity differs. These results demonstrate that exposure to illicit or prescribed opioids during pregnancy may disrupt neurodevelopment and contribute to altered dopamine system function in adulthood.

## Synthesis

Reviewing Editor: Julie Bakker, University of Liege Decisions are customarily a result of the Reviewing Editor and the peer reviewers coming together and discussing their recommendations until a consensus is reached. When revisions are invited, a fact-based synthesis statement explaining their decision and outlining what is needed to prepare a revision will be listed below. The following reviewer(s) agreed to reveal their identity: NONE. Note: If this manuscript was transferred from JNeurosci and a decision was made to accept the manuscript without peer review, a brief statement to this effect will instead be what is listed below.

Synthesis: the ms has been seen by 2 reviewers and both see merit in the work. However, it will be important to conduct the TTX experiment as suggested by the second reviewer to better explain why the vHipp or PVT were targeted in the first place.

Reviewer 1:

The manuscript provides strong evidence for deleterious consequences of in-utero buprenorphine.

1/ Showing graphs with both sexes combined is confusing at best. The fact that the authors studied both sexes is commendable. Therefore, the graphs should show separate sexes. Showing the combined data is unnecessary. This will highlight the sex differences that are evident in many parameters and will be a real advance in the consideration of sex as a biological variable.

2/ The authors claim to be studying the consequences of in utero opioids in adulthood. However, the age of the animal (60+ days) makes it unclear whether this is an adult study (rats are truly adult after about 3-6 months (see Marinelli for example McCutcheon and, European Journal of Neuroscience, Vol. 29, pp. 997-1014, 2009; Sengupta International Journal of Preventive Medicine, Vol 4, No 6, June, 2013). This point should be addressed early in the manuscript and conclusions should be mitigated throughout the paper.

3/ The choice of PPI as the only behavioral indicator is questionable. Depression/reward-related (e.g., sucrose preference, social interaction) tests would have been more relevant, especially considering that putative DA cells from the VTA were recorded as sole physiological readout.

4/ The TTX experiments are crude, and this reviewer does not understand why hHippV and PVT were targeted.

5/ Finally, from a translational perspective, treating mothers during gestation does not mimic human pregnancy since the third trimester of human pregnancy corresponds to the first two weeks of postnatal life in rats. That too must be clearly specified and discussed appropriately.

Reviewer 2:

This study shows that in utero exposure to oxycodone as well as Buprenorphine a compound commonly used as “opiate substitution treatment” can cause in rats behavioural alterations and dopamine neurons hyperactivity in adulthood.

Overall the question is interesting and the experiment design is appropriate although more details could be presented in the figures to convince readers on the quality of data collection. For example:

- Withdrawal score: How many time were the symptoms (vocalization, hyperactivity, stretching, face washing, and tremors) observed, for how long?

- Population activity: How many cells were detected in total, in each animal? What are the criteria of a spontaneously active dopamine neuron?

Minor point

Buprenorphine usually used to reduced withdrawal symptoms at birth is apparently producing the same or higher withdrawal score in newborn rats than oxycodone (Figure 1). Do the authors found that Buprenorphine is not able to reduce NAS compared to other full agonist opiates? Could the authors comment more on this point in the discussion?

Major point

In the last figure (Figure 4) TTX injections were performed to restore normal dopamine neurons activity in adult rats exposed to oxycodone or buprenorphine in utero. This experiment would require to be also done in saline-treated animals. I think this is crucial in order to test for a simple non-specific effect on population activity driven by vHipp or PVT or to conclude that the pharmacological manipulation specifically restore basal activity of VTA neurons.
